# The Identification and Heterologous Expression of the Biosynthetic Gene Cluster Encoding the Antibiotic and Anticancer Agent Marinomycin

**DOI:** 10.3390/biom14010117

**Published:** 2024-01-16

**Authors:** Emily Abraham, Hannah A. Lawther, Yunpeng Wang, Joseph S. Zarins-Tutt, Gerry Sann Rivera, Chengcang Wu, Jack A. Connolly, Gordon Florence, Matthias Agbo, Hong Gao, Rebecca J. M. Goss

**Affiliations:** 1 Department of Chemistry & BSRC, University of St. Andrews, St. Andrews KY16 9ST, UK; emilyrebeccaabraham@gmail.com (E.A.); jack.connolly@manchester.ac.uk (J.A.C.);; 2 Intact Genomics, St. Louis, MO 63132, USAcwu@intactgenomics.com (C.W.)

**Keywords:** antibiotic, VREF, MRSA, melanoma, synthetic biology, biosynthetic gene cluster, heterologous expression, actinomycete, marine natural product

## Abstract

With the rise in antimicrobial resistance, there is an urgent need for new classes of antibiotic with which to treat infectious disease. Marinomycin, a polyene antibiotic from a marine microbe, has been shown capable of killing methicillin-resistant *Staphylococcus aureus* (MRSA) and vancomycin-resistant *Enterococcus faecium* (VREF), as well as having promising activity against melanoma. An attractive solution to the photoprotection of this antibiotic has been demonstrated. Here, we report the identification and analysis of the marinomycin biosynthetic gene cluster (BGC), and the biosynthetic assembly of the macrolide. The marinomycin BGC presents a challenge in heterologous expression due to its large size and high GC content, rendering the cluster prone to rearrangement. We demonstrate the transformation of *Streptomyces lividans* using a construct containing the cluster, and the heterologous expression of the encoded biosynthetic machinery and production of marinomycin B.

## 1. Introduction

**Figure 1:**
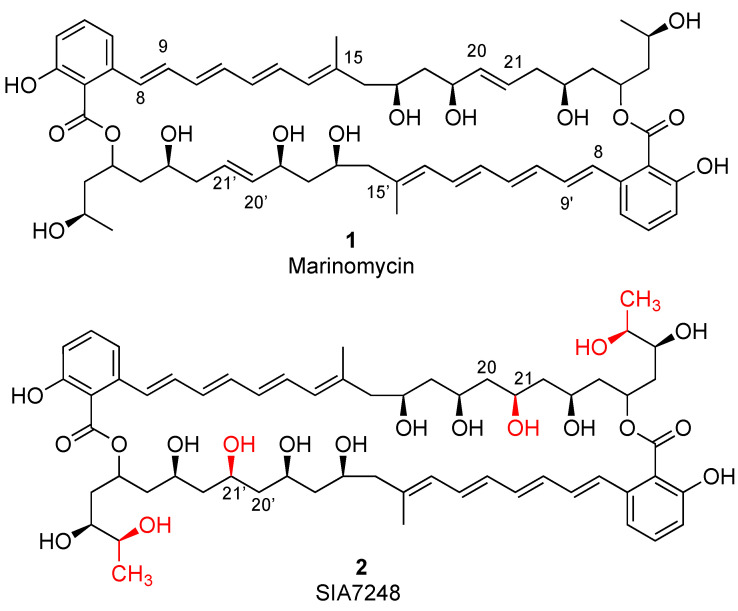
Marinomycin (**1**), and SIA7248 (**2**). Key differences between the metabolites are highlighted in red.

The quest for new antibacterial compounds is urgent. Since the end of the 20th century, over 13 million lives worldwide have been claimed each year due to infectious diseases [1]. A hidden pandemic linked to antibiotic resistance is predicted [2]. Conversely, very few new antibiotics have been introduced to the market in the last 45 years, with this limited number falling into only five new structural classes: oxazolidinones, lipopeptides, mutilins, tiacumicins, and diarylquinolines [3]. It is important to diversify the current antibiotic repertoire and continue to develop compounds from alternative classes; one such class with potential for further development are polyenes. Polyenes are a crucial family of antibiotic compounds, notably containing nystatin, natamycin, amphotericin B, and candicidin [4]. However, many polyenes have poor photostability and can isomerise around the polyene motif [5]. Marinomycins are a fairly recently discovered class of polyene antibiotic (Figure 1), consisting of three stereoisomers: marinomycin A (*trans* ∆^8,9^, *trans* ∆^8′,9′^), marinomycin B (*cis* ∆^8,9^, *cis* ∆^8′,9′^), and marinomycin C (*trans* ∆^8,9^, *cis* ∆^8′,9′^). Marinomycin A has been shown to have excellent antibiotic activity against the notorious pathogen methicillin-resistant *Staphylococcus aureus* (MRSA) and vancomycin-resistant *Enterococcus faecium* (VREF) (both MIC_90_ = 0.13 µM) [6]. As with many polyenes, photostability represents a challenge [7]. Marinomycin A has been shown to have t_1/2_ of 95 s in sunlight and 8 s in UV light [6,8]. Notably, it has been demonstrated that photoisomerization of marinomycin can be prevented through packaging of marinomycin in safe-to-ingest pollen or spore exines, a natural packaging material with FDA approval [8]. Due to this finding, there is potential to develop marinomycin as an antibiotic [7].

Identification and heterologous expression of BGCs encoding natural products of interest are important. They enable the gene cluster to be moved into a more readily fermentable and genetically tractable host, opening up future opportunities for controlling and enhancing gene expression, and thus the production or modification of the BGC to generate analogues. Heterologous expression can be significantly hampered by large, GC-rich and repetitive BGCs. Such BGCs are hard to clone and can readily succumb to recombination events.

In this study, we sought to first identify the genes involved in marinomycin biosynthesis, and to then explore their heterologous expression in a strain that was more amenable to fermentation, and with increased genetic tractability compared to the natural host “*Marinispora*” CNQ-140. Though we have established transformation of this strain, transformation rates are low. Such a heterologous expression system could provide a valuable platform for future studies in engineering enhanced production and for a system which could potentially use combinations of synthetic chemistry and synthetic biology to dial into new analogues of this promising antibiotic. The BGC encoding this type 1 polyketide polyene was likely to be large. Furthermore, GC-rich and likely highly repetitive modules would render the BGC particularly challenging, as it would be prone to homologous recombination. This BGC would thus also provide a useful test-bed with which to challenge the current limits of cloning and heterologous expression.

## 2. Materials and Methods

### 2.1. Microorganisms

*E. coli* strain DH10B-T1 (F-*mcrA* Δ(*mrr-hsdRMS-mcrBC*) φ80*lacZ*ΔM15 *ΔlacX74 nupG recA1 araD139* Δ*(ara, leu)7697 galU galK rpsL endA1* λ^−^ *tonA*) was used for routine plasmid propagation and subcloning. *E. coli* strain ET12567/pUZ8002 was used as the donor host in plasmid conjugations from *E. coli* to *Streptomyces,* as described previously [9]. *E. coli* BW25113/pIJ790 was used as the host for Red/ET-mediated recombination to carry out insertional inactivation of enzymes [10].

Various *Streptomyces* hosts were tested in this work, including *Streptomyces lividans* ΔactΔred, a gift from Michael Thomas lab, University of Wisconsin, Madison; *S. lividans* TK24 (str-6 SLP2^−^ SLP3^−^) [11]; and *Streptomyces albus* J1074 [12]. “*Marinispora*” CNQ-140 was a gift kindly provided by Prof. William Fenical (Scripps Institution of Oceanography, San Diego, CA, USA).

### 2.2. Genomic DNA Isolation and Analysis

“*Marinispora*” CNQ-140 was grown in 50 mL tryptone casitone glucose–marine broth (TCG marine) at 28 °C, 200 rpm, for 48 h. Genomic DNA was isolated from well-dispersed liquid cultures. Firstly, the cultures were pelleted by centrifugation (Thermo Scientific IEX CL30R centrifuge (ThermoFisher Scientific, Cambridge, UK) with T41 swinging bucket rotor, 4000 rpm, 10 min). The supernatant was then discarded, and the pellet was resuspended in TE buffer. The cells were then lysed by the addition of 10 mL lysis solution (0.1 M NaCl, 10 mM Tris-HCl, 50 mM EDTA, pH 8.0) and 10 mg lysozyme. The mixture was frozen in liquid nitrogen and then heat-shocked at 60 °C. This freeze–thaw process was repeated four times. An equal volume of phenol-chloroform isoamyl alcohol (25:24:1) was then added, and this solution was shaken to mix thoroughly. The solution was then centrifuged (3000 rpm, 10 min), after which the top aqueous layer was carefully removed by pipetting. An equal volume of chloroform-isoamyl alcohol mixture (24:1) was added to the aqueous phase, and centrifugation was repeated, after which the top layer was again removed by pipetting. The DNA was then precipitated with a 0.8 volume of isopropanol, and the gDNA was ‘spooled’ out when possible and washed with 70% ethanol. When it was not possible to visualise the DNA to ‘spool’ it out, the sample was centrifuged, and the resulting pellet was washed with 70% ethanol. Air-dried gDNA was dissolved with TE buffer.

### 2.3. Insertional Inactivation of HMGS (3-Hydroxy-3-Methylglutaryl-CoA Synthase)

To confirm that the predicted BGC is responsible for marinomycin’s biosynthesis, one of the feature enzymes, HMGS, was inactivated in “*Marinispora*” CNQ 140 by the PCR-targeting system [10] as follows. The primer pair of HMG-LF/LR (Table S1) was used to amplify the replacement cassette from plasmid pIJ773. The PCR generation of the replacement cassette with the expected size (~1.5 kb) was verified by agarose gel electrophoresis (Figure S1). The primer pair of HMG-NF/HMG-NR (Table S1) was used to amplify *hmgs* gene and flanking sequences from the genomic of “*Marinispora*” CNQ-140. The PCR product with the expected size (~3.3 kb, including up- and down-stream flanking fragments, ~1.1 kb each) was verified by agarose gel electrophoresis (Figure S2). The amplified *hmgs* gene was purified using Wizard^®^ SV Gel and a PCR Clean-up System (Promega, Hampshire, UK), and ligated to pJET 1.2/blunt per the manufacturer’s suggestion, to construct pJET 1.2-hmgs. pJET 1.2-hmgs was confirmed by PCR using HMG-NF and HMG-NR as primers (Figure S3) and sequencing data.

*E. coli* BW25113/pIJ790 was transformed by pJET 1.2-hmgs. Then, BW25113/pIJ790/pJET 1.2-hmgs was plated onto selective LB agar (25 μg/mL chloramphenicol and 100 μg/mL ampicillin) and incubated at 30 °C overnight. Single colonies of the overnight growth were inoculated into fresh LB broth (10 mL, with the same antibiotics) and incubated overnight at 30 °C and 200 rpm. The overnight cultures were diluted 100-fold in SOB (10 mL, same antibiotics) containing L-arabinose (10 mM), and incubated at 30 °C and 200 rpm until an OD600 of ~0.4 was reached. The cultures were centrifuged (2700× *g*, 10 min, 4 °C) and the pellets were washed twice with chilled sterile 10% aqueous glycerol (10 mL). The cells were resuspended in the residual 10% (*v*/*v*) aqueous glycerol (50–100 μL). The cell suspension was mixed gently with the chilled PCR amplified gene replacement cassettes (~100 ng) in chilled 0.2 cm electroporation cuvettes. Electroporation was carried out using a BioRad GenePulser II (2.5 kV, 4.5–4.9 millisecond, Bio-Rad, Hertfordshire, UK). Immediately, chilled LB broth (1 mL) was added to the electroporated cells and incubated at 37 °C and 200 rpm for 1 h, and then cultures were plated onto LB agar (100 μg/mL apramycin, 100 μg/mL ampicillin) and incubated overnight at 37 °C. Colonies (5–10) were picked at random and inoculated into LB broth (10 mL, 100 μg/mL apramycin, 100 μg/mL ampicillin) and incubated overnight at 37 °C to prepare the plasmid pHG1 (in which the *hmgs* gene was replaced by *acc(3)IV*-*oriT*, Figure S4) by alkaline lysis. pHG1 was sequenced using pJET1.2 sequencing primers, confirming its sequence and constriction.

The plasmid pHG1 was used to transform chemically competent *E. coli* ET12567/pUZ8002 (non-methylating). *E. coli* ET12567/pUZ8002/pHG1 and the “*Marinispora*” CNQ 140 mycelium were mixed onto MS agar plates (complemented with instant ocean) containing 10 mM MgCl_2_ and incubated at 28 °C for 20 h. The MS plates were overlaid with water (1 mL) containing nalidixic acid (0.5 mg) and apramycin (1.25 mg) and incubated at 28 °C until sporulation. MS plates of control *E. coli* ET12567/pUZ8002 and “*Marinispora*” CNQ 140 (overlaid with nalidixic acid only) were also incubated at 28 °C for monitoring successful conjugation. The resultant exconjugant colonies were replicated on MS agar (complemented with instant ocean) containing nalidixic acid (25 μg/mL) and apramycin (50 μg/mL), and incubated at 28 °C until sporulation. Successful gene replacement was confirmed by PCR using total gDNA prepared from the mutant strains as PCR templates (Figure S5) and sequencing data. The new strain was named CNQ 140/*hmgs*^−^ (*hmgs::aac(3)IV-oriT*).

### 2.4. Genetic Complementation

The *hmgs* genes were PCR amplified using the primer pair of HMG-CF/CR (Table S1) from the genomic DNA of “*Marinispora*” CNQ-140 and purified using Wizard^®^ SV Gel and PCR Clean-up System according to the manufacturer’s instructions. Purified PCR products were digested by the appropriate restriction enzymes (NdeI and HindIII) and ligated into pIJ10257 linearised by the same enzyme pair under the control of the erythromycin resistance promoter *ermEp*. The ligation products (6 μL) were transformed into *E. coli* DH10B-T1 competent cells, plated onto DNA agar containing hygromycin B (50 μg/mL), and incubated overnight at 37 °C. Overnight LB broth cultures (10 mL) of the resultant colonies with addition of hygromycin B (50 μg/mL) were used for plasmid preparation.

The purified plasmid, pHG2 (Figure S6), was verified by PCR (Figure S7) and sequencing data, then transformed into *E. coli* ET12567/pUZ8002. It was then conjugated with the mycelium of strain CNQ 140/*hmgs*^−^ (*hmgs::aac(3)IV-oriT*). The exconjugants were confirmed by PCR (Figure S8).

### 2.5. Cosmid and BAC Library Generation and Screening

The cosmid vector SuperCos 1 was used in the construction of the genomic library of “*Marinispora*” CNQ-140, using a Gigapack III XL Packing Extract kit (Agilent, Santa Clara, CA, USA), according to the manufacturer’s instructions. The cosmid library was screened using three sets of primers (Clu36, Clu18 and Clu11, Table S1), which were designed to amplify partial fragments of *marE*, *marG* and *marI*. From this screening, it was revealed that the proposed marinomycin BGC was spread over three cosmids; the cosmids were then Sanger sequenced to determine the full sequence of the proposed marinomycin BGC. Due to rearrangements occurring because of the high GC content and multiple repeats within the cluster, multiple ligation strategies failed to yield the full construct of the BGC, and thus a BAC library of “*Marinispora*” CNQ-140 was constructed.

Large plasmids (over 20 kb) such as pIB139_CNQ140 were extracted from a 500 mL volume. The culture was pelleted by centrifugation and the pellet was resuspended in 2 mL of TE buffer. Next, 3 mL of lysis buffer was added, and the sample was incubated at room temperature for 5 min. Then, 9 mL of neutralization solution was added, and the sample was incubated at −80 °C for 30 min. The sample was then thawed and centrifuged (4000 rpm, Thermo Scientific IEX CL30R centrifuge with T41 swinging bucket rotor at 4 °C for ten minutes). The supernatant was then extracted using one volume of phenol-chloroform isoamyl alcohol (25:24:1). The top, aqueous phase was carefully removed and one volume of chloroform-isoamyl alcohol mixture (24:1) was added to the aqueous phase. This was centrifuged again at 4000 rpm for 10 min at 4 °C. The supernatant was decanted into a clean microcentrifuge tube, and the DNA was precipitated to concentrate it.

Glycerol stocks of the BAC library were used to inoculate 2 mL cultures in 96-deep well plates with the appropriate antibiotic, and the cultures were incubated at 37 °C overnight. Each well corresponded to a separate *E. coli* colony containing a separate BAC vector and insert. The next day, the cultures were pelleted by centrifugation, and the plates were inverted over a paper towel to remove the growth medium, while ensuring the bacterial pellet was not lost. The pellet was then resuspended in 50 uL of ice-cold buffer 1 (50 mM D-glucose, 10 mM EDTA, 25 mM Tris-HCl, pH 8.0). The cells were then lysed by the addition of lysis buffer, followed by incubation on ice for five minutes. Next, 75 uL of ice-cold neutralisation solution was added and mixed gently. The cultures were then incubated on ice for 5 min before being centrifuged for 15 min at 4 °C. Next, the 150 uL of supernatant from each culture was removed and transferred to a fresh 96-deep well plate, and 200 uL of ice cold 2-propanol was added to each well to precipitate the DNA. The plates were then centrifuged again at 4 °C to pellet the DNA. After this, the plates were inverted to remove the 2-propanol and 200 uL of 70% ethanol solution was added, followed by gentle agitation. The plates were then centrifuged for the last time to pellet the DNA at 4 °C, after which the ethanol was removed and the pellets were left to completely dry. Finally, each DNA pellet was resuspended in 30 uL of TE buffer, and the DNA was used for PCR screening (Figures S9–S12).

### 2.6. Strain Transformations with BAC Construct

A variety of conditions were screened to lead to the highest efficiency and successful conjugates of these BAC plasmids: #4, #6 and #9. These conditions varied from solid media: TSB vs. MS vs. soya flour media, helper plasmids, Streptomyces strains including *S. lividans* and *S. albus*, and conjugation times. With all these conditions varied, the procedure stated below can be applied to all combinations of conditions. *E. coli* BAC cells were grown in LB with antibiotic selection. All *E. coli* cells and strains were grown at 37 °C to an optical density of 0.4–0.6, spun down and washed with fresh LB to remove residual antibiotics. Streptomyces spores were thawed at 10^9^ cells/mL density. Cells were mixed together and plated on soya flour solid media and grown for 16–18 h at 28 °C. The plates were then overlaid with nalidixic acid (25 µg/mL) and apramycin (50 µg/mL), and incubated again at 28 °C until sufficient spores were produced. Successful exconjugants were then picked, grown in liquid TSB media with antibiotic selection for 48 h. Mycelia in the liquid broth were then properly resuspended and plated onto Soya Flour solid media with antibiotic selection and grown for 5–7 days. Spores were collected and frozen in −80 °C, then the exconjugants were confirmed by PCR using two primer sets of Clu18 and Clu36 (Table S1), which were also used in the screening of cosmid and the BAC library.

### 2.7. Analysis of Marinomycin Production

Strains were inoculated onto MS agar plates (mannitol 20 g/L, soya flour 20 g/L, agar 20 g/L, and apramycin 50 μg/mL) at 28 °C for 4 days. Single colonies were picked and used to inoculate 5 mL of appropriate ISP2 for 3 days. Then, 1 mL cultures were inoculated into 50 mL ISP2 media with 50 μg/mL apramycin and incubated for 7 days (28 °C, 200 rpm) before pelleting by centrifugation (4000 rpm, 10 °C, 30 min). The supernatant was used for UPLC analysis on an Acquity UPLC H class system (Waters, Wilmslow, UK) or LC-HRMS analysis on a Termo Scientific Velos Pro/Orbitrap Velos Pro (ThermoFisher Scientific, Cambridge, UK).

UPLC analysis was carried out using a Phenomenex kinetex 2.6 μm Phenyl–Hexyl 75 × 2.10 mm column (Phenomenex, Macclesfield, UK). The column was held at 60 °C and an injection volume of 5 μL, and a flow rate of 600 μL/min was used for all samples. UPLC analysis was carried out with 0.1% formic acid in water and methanol. The following gradient was used: 0.00–0.20 min 5% methanol, 0.2–1.00 min 5–65% methanol, 1.00–2.00 min 65–80% methanol, 2.00–4.00 min 80% methanol, 4.00–4.45 min 80–5% methanol, 4.45–5.00 min 5% methanol. UV absorbance was measured at 360 nm at a resolution of 2.4 nm.

LC-HRMS was carried out with an H-ESI source and a Themo Scientific Diones UltiMate 3000 RS chromatography system (ThermoFisher Scientific, Cambridge, UK). The HPLC system was equipped with a Waters XBridge C18 3.5 μm 2.1 × 100 mm column at 40 °C. A 5 μL injection volume was used for all samples. HPLC analysis was carried out with 0.1% formic acid in water and acetonitrile with a flow rate of 350 μL/min. The following gradient was used: 0.00–1.50 min 5% acetonitrile, 1.50–8.00 min 5%–95% acetonitrile, 8.00–10.00 min 95% acetonitrile, 10.00–10.50 min 95–5% acetonitrile. UV absorbance was measured between 220–800 nm at 2 nm resolution. The first minute of the run was diverted to waste. After 1 min, the eluent was passed to the H-ESI source. The HRMS was set up with the following parameters: Negative ionisation mode using a 250 °C heater temperature, 350 °C capillary temperature, 35 (arb) sheath gas flow, 10 (arb) aux gas flow, 2 (arb) sweep gas flow, and a 3.5 kV spray voltage. A background ion corresponding to the [M+H]^+^ of *n*-butly benzenesulfonamide was used as a lock mass for internal scan-by-scan calibration.

## 3. Results and Discussion

### 3.1. Identification and Validation of the Marinomycin BGC

**Figure 2:**
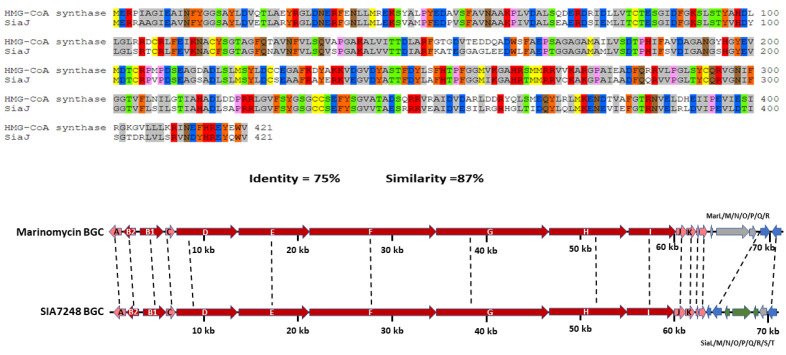
Comparison of the very similar marinomycin and SIA7248 BGCs. (**Top**) the validated HMGS cassette (marJ) contained in the predicted BGC for marinomycin. (**Bottom**) known BGC for SIA7248. Links with arrows show genes common to both biosynthetic pathways.

**Figure 3:**
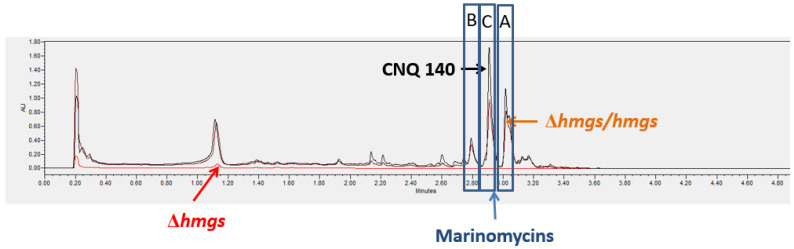
Ion-extracted LC-MS trace of the cultures of marinomycins’ production of WT and mutant strains. Black: WT CNQ 140; red: Δ*hmgs*, the *hmgs* knock-out mutant (CNQ 140/*hmgs*^−^ (*hmgs::aac(3)IV-oriT*), no marinomycins produced); orange: Δ*hmgs*/*hmgs*, a genetic complementation strain, restoring the production of marinomycins.

The 72 Kb BGC for marinomycin was initially putatively identified by members of our team in 2012 through analysis of 454 sequence data (Genbank Access No.: OL688631). A genome scan of “*Marinispora*” CNQ-140 was undertaken. With the “*Marinispora*” CNQ-140 genome size predicted to be approximately 7 Mb, the available data corresponded to a 40-fold coverage. Assembly of the sequence reads into 1284 contigs enabled the construction of a database for in silico screening. The distinctive C15/15′ pendent methyl at the ß position of a polyketide is indicative of the operation of HMGS (MarJ in Figure 2). The candidacy of this gene was confirmed through insertional inactivation of the *marJ* with an apramycin resistance cassette. Production of marinomycin by the strain could be restored by genetically complementing with a further copy of this gene (Figure 3). The HMGS inactivated strain, Δ*hmgs* (represented by a red line in the figure), does not produce any of the three marinomycins produced by the wild type (WT) (represented by a black line in the figure). It is worth noting that there are four nucleotides (GTGA) overlapping between *marJ* and the downstream *marK*, but the inserted cassette brings the four nucleotides back to restore the function of *marK*, so all the other enzymes are not interfered by the *in-frame* deletion of *marJ*. When the *marJ* gene is complemented back into the Δ*hmgs* strain, Δ*hmgs*/*hmgs* (represented by an orange line in the figure), production of all three of the marinomycin compounds is restored.

The identity of the BGC was further confirmed through comparison with the subsequently published BGC for the marinomycin analogue SIA7248 [13] (Figure 1), a BGC that was later found, curiously, in the human oral cavity [14]. SIA7248 shares many common features with the dimeric structure of marinomycin, such as its constituent polyketide chains. The few differences reflect that the dehydration event that gives rise to the C20, a 21 (20′, 21′) double bond in marinomycin, does not occur for SIA7248, and that instead a hydroxyl remains at C44. Moreover, each polyketide chain of SIA7248 is one carbon longer, reflecting the proposed use of lactate rather than acetate as the starter unit of SIA7248. Though SIA7248 also represents a challenging BGC in terms of its high GC content and the repetitive nature of its modules, and has not yet been heterologously expressed; to the best of our knowledge, its activity profile is not yet known, and marinomycin remains the focus of our studies due to its potent activities.

### 3.2. Comparison of the Marinomycin and SIA7248 BGCs

A longer read sequence analysis was performed using MinION nanopores. This enabled a detailed analysis of the 72 Kb marinomycin BGC and comparison of the BGC with SIA7248, a metabolite identified whilst this study was ongoing. Though the sequence identity of the SIA7248 and marinomycin BGC is 78%, it is notable that both the organisation and orientation of the 14 open reading frames encoding the molecules are the same (for a full analysis, see SI Section S7, Tables S3 and S4).

The genes with the greatest differences may be found at the beginning of the biosynthetic pathway, reflecting a difference in the starter units used. In SIA7248 biosynthesis, *siaD* uses a glycerol-derived lactate as the starter unit, whereas in marinomycin biosynthesis, an acetate starter unit is used. Phylogenetic analysis (SI Section S8) groups the KS domain in module 1 of the marinomycin BGC (marD_KS1) with the first KS in the biosynthesis of anthracimycin (Atc), disorazol (Dis), and macrolactin (Mln). These three polyketide synthases (PKSs) lack a loading module [15]. Module 5 in marinomycin BGC contains an active Dehydratase (DH) domain, generating the C20, C21 double bond, lacking in SIA7248.

**Scheme 1:**
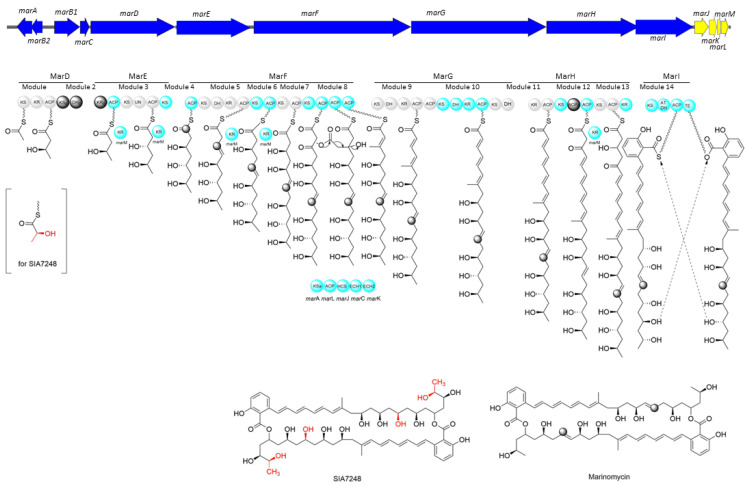
(**Top**) BGC for the production of Marinomycin A. (**Bottom**) Proposed biosynthetic pathway for the production of Marinomycin. KS, DH and KR in module 2 and the first ACP in module 12, indicated by grey shadowed circles, are inactive based on bioinformatics analyses (details of domains’ analysis can be found in SI Section S8, Figures S15–S20). UN in module 3 is tentatively referred to as a UN domain, because no function can be predicted for this whole domain using classical PKS domain bioinformatics analysis. Key differences between SIA7248 and marinomycin are highlighted in red.

As with *siaD*-*siaH* or *marD*-*marH* (encoding modules 1–13 of the SIA7248 and marinomycin biosynthetic machineries, there are no module-embedded acyl transferase (AT) domains, with both systems belonging to trans-AT type I PKS. *siaB1* has been shown to encode a protein which acts as an AT domain as it iteratively loads malonyl-CoA extender units in *trans* onto the acyl carrier proteins (ACPs) of SiaE-SiaH [13]. A proposed biosynthetic scheme is presented (Scheme 1).

### 3.3. Overcoming Challenges Presented by a Large and Sequence-Repetitive Polyene BGC in Enabling Cloning and Heterologous Expression

The high GC content of this organism (74%), in combination with series of high-similarity regions of DNA within the large BGC (72 Kb), presents challenges in cloning and heterologous expression, as there is a high propensity toward homologous recombination and rearrangement [16]. The PKS-encoding genes identified in the BGC with the highest likely identities (as identified by function) were therefore compared to each other. These % identities are shown in Table 1.

Table 1, illustrates that the PKS-encoding genes *marE*-*I* have a fairly high percentage identity across their long open reading frames, indicating propensity for potential recombination, thus making heterologous expression very challenging. All other genes in the cluster showed low identity with each other.

In the first steps toward heterologous expression of this challenging BGC, a cosmid library from “*Marinispora*” CNQ-140 gDNA was generated, and 1200 members picked and validated. A set of three primer pairs, designed to amplify the KS domain from modules 4 and 10, and the ACP domain from module 14 were used in parallel with primers that had been used in the preparation of the ∆*hmgs* mutants to enable a PCR-based screening of the cosmid library for members carrying genes associated with the marinomycin BGC. From candidate cosmids identified as carrying genes associated with the biosynthesis of marinomycin, a set of three cosmids could be identified containing the termini and the middle of the PKS. These three cosmids were then Sanger sequenced, containing a 103 Kb sequence length which contained the approximately 72 Kb marinomycin BGC. A multitude of different cloning, ligation and Gibson assembly strategies were pursued to assemble the full BGC, and although several at first sight appeared to have worked, upon analysis by PCR, restriction digestion, or sequencing, all potential marinomycin BGC constructs were seen to have undergone rearrangement. A different approach was thus needed. Instead, to ensure that all genes were captured within one construct without rearrangement, a simpler approach was pursued, which avoided the need for assembly. Whereas cosmids can contain DNA fragments up to ~42 Kb, BAC inserts can be much larger (200 Kb) [17], enabling the potential capture and cloning of a fragment of gDNA carrying the full marinomycin BGC.

To complement the cosmid library, a BAC library with an average insert length of 145 Kb was generated, and 2688 members picked. An identical PCR screening was applied, and three members of the BAC library were identified as carrying the full PKS component of the BGC.

Different actinomycetes were screened for resistance to marinomycin (Table S2), and *Streptomyces lividans* and *Streptomyces albus* were selected as potential heterologous expression hosts. Stable exconjugants of *S. lividans* could be achieved, though not *S. albus.* The same PCR primers that had been used for identification of the BAC were used to confirm the presence of the full BGC within the heterologous host.

### 3.4. Detection of Marinomycin by LC-MS Analysis

**Figure 4:**
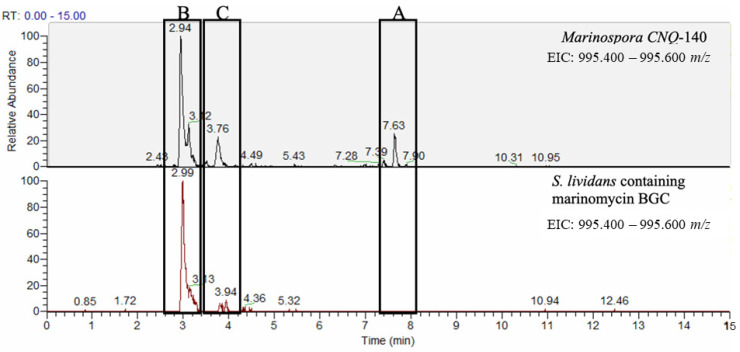
Grey: the natural producer “*Marinispora*” CNQ-140 extracted supernatant EIC shows marinomycins A, B, and C. White: the heterologous host *S. lividans* extracted supernatant EIC shows marinomycins B and C. Top grey: natural producer, marinomycin B, retention time (RT) 2.94 min, *m*/*z* 995.50 [M−H]^−^. Bottom white: heterologous host *S. lividans,* marinomycin B, RT: 2.99 min *m*/*z* 995.50 [M−H]^−^. Details can be checked in SI Section S5, Figures S13 and S14.

The transformants were cultured and the supernatant was taken and diluted 1 in 10 with 50:50 MeOH:H_2_O, and analysed by negative mode HPLC-HR-ESI-MS. In parallel, the WT “*Marinispora*” CNQ-140 was analysed for comparison (Figure 4). “*Marinispora*” CNQ-140 samples showed three peaks at *m*/*z* 995.49365 [M−H]^−^, *m*/*z* 995.44971 [M−H]^−^, and *m*/*z* 995.51331 [M−H]^−^ at retention times of 2.94, 3.76, and 7.63 min, respectively, and the heterologous production host showed two peaks at *m*/*z* 995.49176 [M−H]^−^ and *m*/*z* 995.44183 [M−H]^−^ at a retention time of 2.99 and 3.94, respectively, indicating that indeed marinomycin is heterologously produced.

## 4. Conclusions

Marinomycins are promising polyene antibiotics and anticancer agents. The biogenesis of this metabolite is encoded by a 72 Kb biosynthetic gene cluster. Heterologous expression of such a large and GC-rich biosynthetic gene cluster is very challenging, but possible (after a 10-year effort!). Whilst such a system is not amenable to cloning and ligation of multiple cosmids due to predisposition to recombination events and rearrangement, heterologous expression can be achieved through transforming suitable heterologous hosts with BACs. Heterologous expression can be used for the production of marinomycin. The ability to heterologously express the marinomycin BGC in a readily manipulable streptomycetes host strain paves the way for improved fermentative production and genetic interrogation and manipulation of the pathway encoding this potent and new structural class of antibiotic. With heterologous expression and production now in place, and a clinically appropriate system for photoprotection and delivery identified, it may be possible to advance this potentially valuable antibiotic and anticancer agent.

## Figures and Tables

**Table 1:** A comparative study of % identity between PKS-encoding genes responsible for polyene biosynthesis.

	*marD*	*marE*	*marF*	*marG*	*marH*
*marE*	51.13				
*marF*	56.15	47.60			
*marG*	55.40	45.97	52.01		
*marH*	56.03	52.81	50.56	50.76	
*marI*	51.27	54.09	44.83	46.62	51.59

## Data Availability

The data presented in this study are available in the article and Supplementary Material.

## Supplementary Materials

The following supporting information can be downloaded at: https://www.mdpi.com/article/10.3390/biom14010117/s1.
