# Supplementary material for: The Identification and Heterologous Expression of the Biosynthetic Gene Cluster Encoding the Antibiotic and Anticancer Agent Marinomycin
Source: Biomolecules. 2024 Jan 16;14(1):117. doi: 10.3390/biom14010117 (PMC10813093; doi:10.3390/biom14010117)
Supplement: Supplementary file 1 [file biomolecules-14-00117-s001.zip › biomolecules-2784685-supplementary.pdf]

biomolecules

MDPI

Supporting Information

The Ientification and Heterologous Expression of Biosynthetic Gene Cluster Encoding the Antibiotic and Anticancer Agent: Marinomycin

Emily Abraham [1], Hannah A. Lawther [1], Yunpeng Wang [1], Joseph Zarins Tutt [1], Gerry Sann Rivera [2], Charles Chengcang Wu [2], Jack Connoly [1], Gordon Florence [1], Matthias Agbo [1], Hong Gao [1,*] and Rebecca J. M. Goss [1,*]

[1] Department of Chemistry & BSRC, University of St Andrews, St Andrews, KY16 9ST, United Kingdom
[2] Intact Genomics, St Louis, MO 63132, USA
* Correspondence: h.gao@tees.ac.uk (H.G.); rjmg@st-andrews.ac.uk (R.J.M.G.)

Contents

1 General Experimental

2 Insertional Inactivation and Complementation of HMGS

3 BAC Library Generation and Screening

4 Testing of Strains for Susceptibility to Marinomycin A

5 Growth and Analysis of Heterologous Host and Marinomycin Production

6 Feeding of propionate, 3-fluoropropionate, 4-fluorobutyrate to the WT and heterologous expression strain do not give rise to any observable further products

7 MinION Sequencing, and BGC Analysis

8 Alignments and Branching Analysis

1. General Experimental

Microbiology work was carried out in a Faster BH-EN 2004 Laminar flow cabinet. Cultures were grown from spore stocks in 25% glycerol stored at -20°C or mycelial stocks in 25% glycerol stored at -80°C. Media was autoclaved before use at 121°C load temperature and 124°C chamber temperature for 20 minutes. Cultures were incubated in a New Brunswick Scientific I 26 or Innova 44 incubator shaker with a 2.5 cm orbit diameter. All primers used were listed in Table S1.

Table S1. Primers used in this study

Name	Sequence 5′-3′	Used for
pIB139_AdaptationCNQ (Forward)	GCATGCCGTCCGCGCTGGCAATGTCCACGGTGAACGCCGTCCAATTCCACACAACATACGAG	Adaption of pIB139 to contain homologous ends to Marinomycin BGC (direct cloning approach)
pIB139_AdaptationCNQ (Reverse)	CGTTTCACTCCGGGGCAGACCGCCGGCACACACCCTCATCCAACTTCTCGACAGACGTAGATC	
HMG-NF	GGACCAGGAGATGGGCGACT	Amplification of *hmgs* gene from gDNA, plus up- and down-stream flanking sequence
HMG-NR	GCGATGATCCGGACGATGTC	
HMG-LF	ATGACCGGCGGGAACGCGAACCTGAGGAGAAACGCAATGATTCCGGGGATCCGTCGACC	PCR targeted gene replacement
HMG-LR	CGCCGGGGAGCGGCTCGGTGACGGTGACGACCTGGCTCATGTAGGCTGGAGCTGCTTC	
Hkt-F	CGAGGAACTGCTGGACGG	Verification of gene knock-outs
Hkt-R	CGGTGAAGGTGTTCTTGTGC	
HMG-CF	AGGAGTACATATGGAACGGCCCATCGCCGGC	Genetic complementation
HMG-CR	CCCAAGCTTCACACCCACTCGTACTCCCGGT	
pJET1.2 forward sequencing primer	CGACTCACTATAGGGAGAGCGGC	Sequencing pHG1
pJET1.2 reverse sequencing primer	AAGAACATCGATTTTCCATGGCAG	
EA1_Screen_CNQ (Forward)	ATACGATGCCGCAAGTTTCT	Screening Marinomycin BGC
EA1_Screen_CNQ (Reverse)	CGTTGGTGTACTGCCACATC	
EO_CNQ	TTCGTTTCGGTGGTATGTGA	

(Forward)		Screening Marinomycin BGC
EO_CNQ (Reverse)	CCACAAGCAGACAGGACGTA	
Clu11 (Forward)	TGGAGAAGGACGCCGAGGAC	Screening Marinomycin cosmid and BGC
Clu11 (Reverse)	CGAGCCGCCGTAGAAGTTGAT	
Clu18 (Forward)	GTGCCCGATGTTGGACTTGACC	Screening Marinomycin cosmid and BGC
Clu18 (Reverse)	GCTTCGACCCGCTCTTCTTC	
Clu36 (Forward)	GAGAACATCGGCATCTTCGTCG	Screening Marinomycin cosmid and BGC
Clu36 (Reverse)	GCAGCGGTGGGTGAAGTGGT	

2. Insertional Inactivation and Complementation of HMGS

The PCR generation of the replacement cassette with the expected size (~ 1.5 kb) was verified by agarose gel electrophoresis (Figure S1).

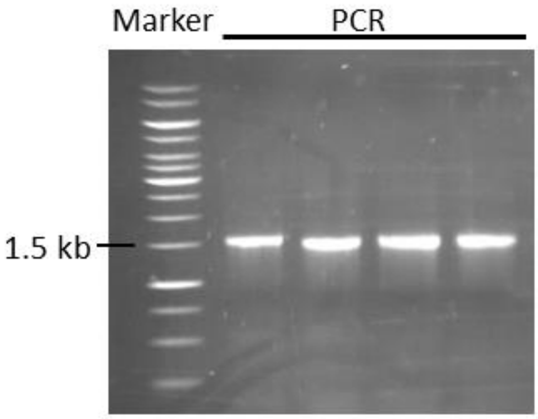

Figure S1: PCR amplification of replacement cassettes for the gene *hmgs* (encoding HMG-CoA Synthase, or named as *marJ*); Marker: GeneRuler 1 kb DNA Ladder (Thermo Scientific, USA).

The PCR product with the expected size (~ 3.3 kb, including up- and down-stream flanking fragment, ~ 1.1 kb each) was verified by agarose gel electrophoresis (Figure S2).

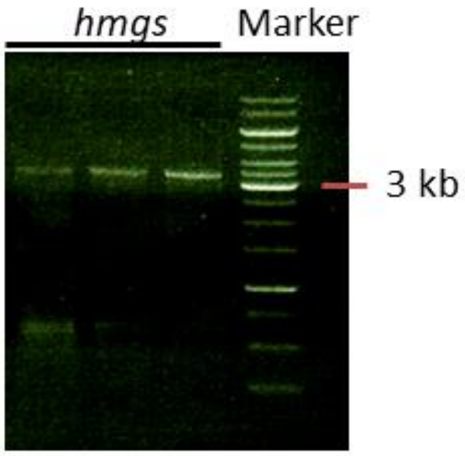

Figure S2: Amplified *hmgs* gene from gDNA of "*Marinispora*" CNQ 140; Marker: GeneRuler 1 kb DNA Ladder.

pJET 1.2-hmgs was confirmed by PCR using HMG-NF and HMG-NR as primers (Figure S3) and sequencing data.

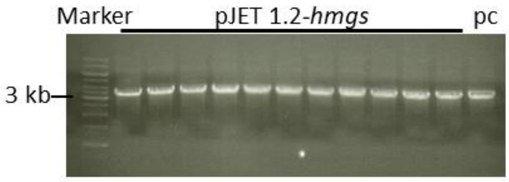

Figure S3: PCR confirmation of pJET 1.2-hmgs.
Lane 1: GeneRuler 1 kb DNA Ladder; Lane 2-12: PCR result from different single colony containing pJET 1.2-hmgs; pc: positive control, using gDNA of "*Marinispora*" CNQ-140 as template in PCR.

The plasmid pHG1 (in which *hmgs* gene was replaced by *acc(3)IV-oriT*, Figure S4) was sequenced using pJET1.2 sequencing primers for the confirmation.

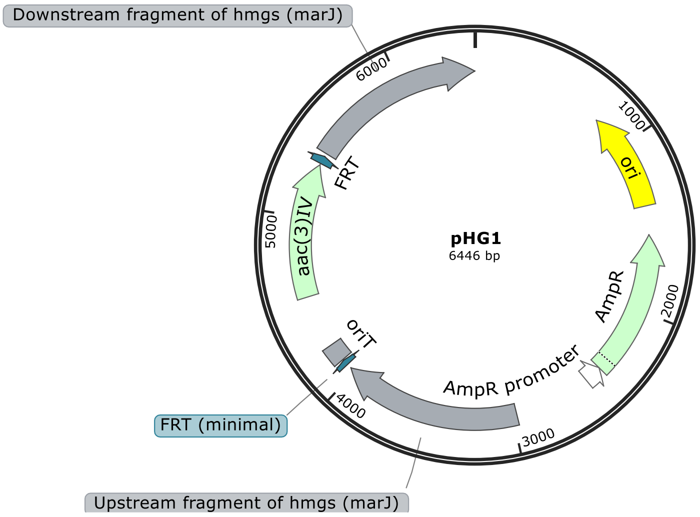

Figure S4. Map of pHG1

Successful gene replacement was confirmed by PCR using total gDNA prepared from the mutant strains as PCR templates (Figure S5) and sequencing data.

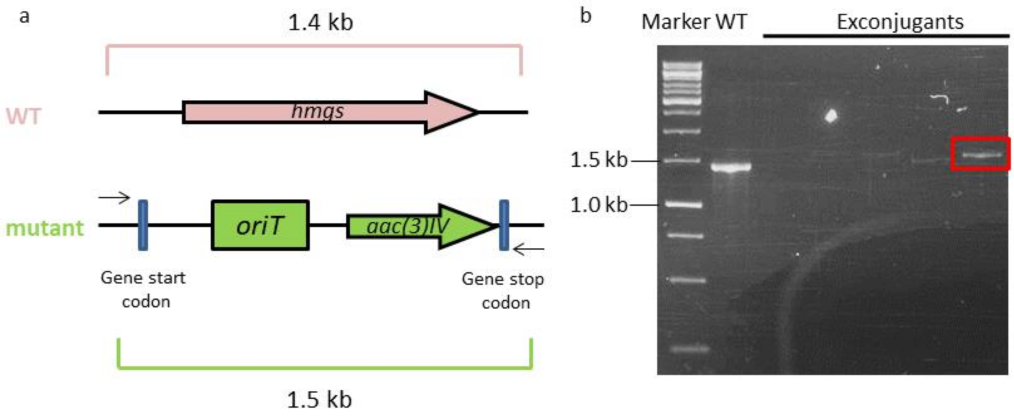

Figure S5: Schematic representation of gene replacement and PCR confirmation.

a. General representation of the gene and replacement cassette, arrows denote primer set (Hkt-F/R) used for confirmation; b. Gel electrophoresis for PCR confirmation. Lane 1: GeneRuler 1 kb DNA Ladder; Lane 2: gDNA from WT strain as template; Lane 3-7: gDNA from different single mutant colonies as template; the one in lane 7 (in red box) was selected for further study, named as CNQ 140/*hmgs*$^-$ (*hmgs::aac(3)IV-oriT*).

The purified plasmid, pHG2 (Figure S6) was verified by PCR (Figure S7) and sequencing data, then transformed into *E. coli* ET12567/pUZ8002, then were conjugated with the mycelium of strain CNQ 140/*hmgs*$^-$ (*hmgs::aac(3)IV-oriT*). The exconjugants were confirmed by PCR (Figure S8).

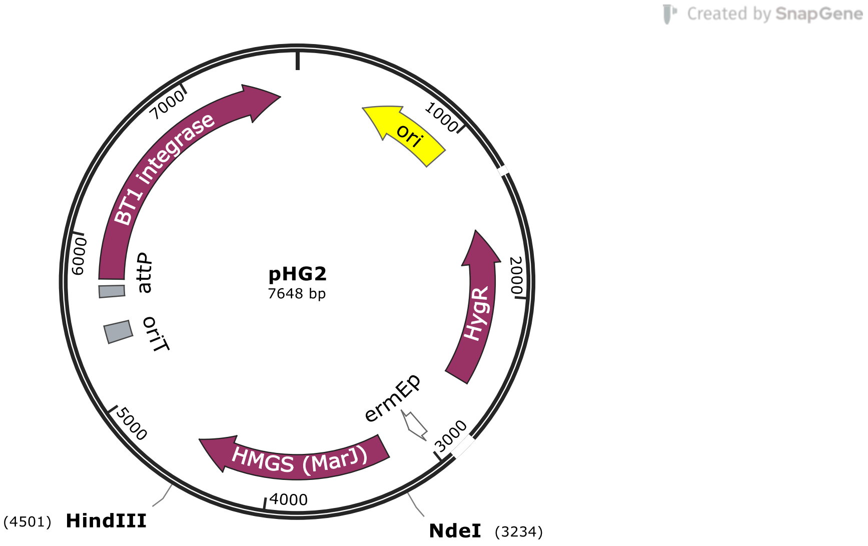

Figure S6. Map of pHG2

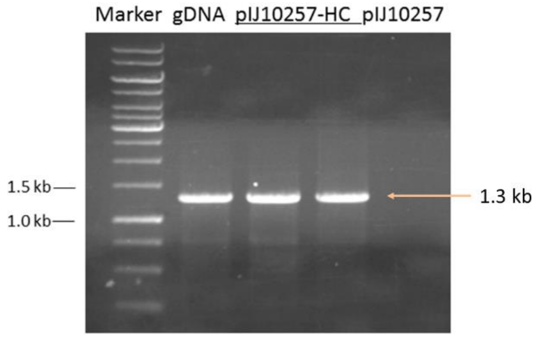

Figure S7: PCR confirmation of pHG2. Lane 1: GeneRuler 1 kb DNA Ladder; Lane 2: Products of expected size (~ 1.3 kb) were amplified from gDNA of "*Marinispora*" CNQ-140, positive control; Lane 3 and 4: Products from pHG2; Lane 5: pIJ10257 was used as template for PCR, negative control.

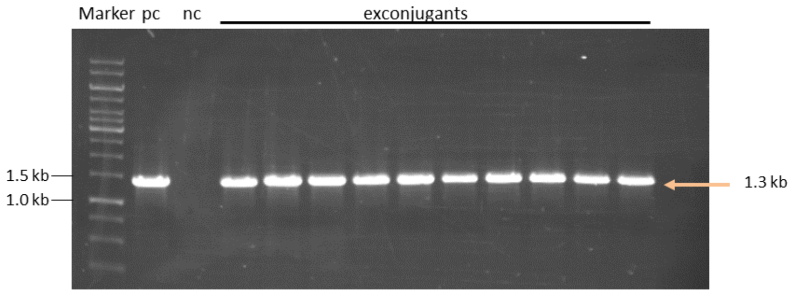

Figure S8: PCR confirmation of conjugants after genetic complementation. Lane 1: GeneRuler 1 kb DNA Ladder; Lane 2: pc, positive control, using gDNA of "*Marinispora*" CNQ 140 as template; Lane 3: nc, negative control, using gDNA of CNQ 140/*hmgs*$^{-}$ (*hmgs::aac(3)IV-oriT*) as template. Lane 4-13: PCR results using exconjugants as template. All the exconjugants picked up are successful transformed with pHG2.

3. BAC Library Generation and Screening

A pooled 'master' BAC DNA plate was first screened with the EA1 primer set, which amplified a 456 bp region near the N terminus of the Marinomycin BGC (Figure S9). For this PCR, the majority of the 96 positions on the pooled DNA plate were positive for the EA1 PCR product, and therefore positions containing the brightest bands were picked for further screening.

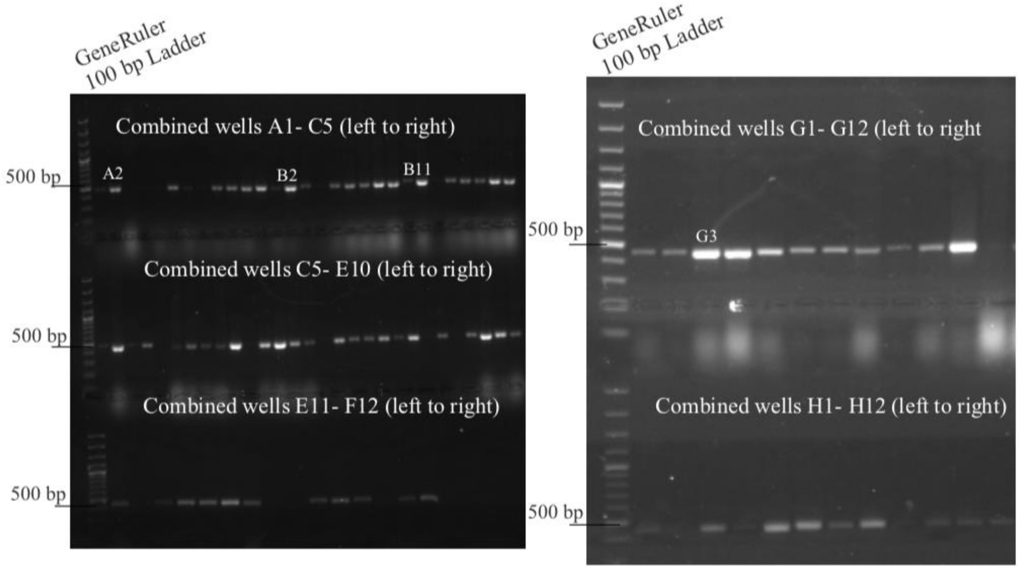

Figure S9. PCR conducted on pooled BAC DNA plate with the EA1 primer set. The correct band size (456 bp) was seen for many of the positions. Positions with particularly bright bands were chosen for further screening: A2, B2, B11 and G3.

Positions A2, B2, B11 and G3 were next individually screened on each of the twenty-eight individual BAC DNA plates with primers that amplified regions at the beginning and end of the BGC (primer sets EA1 and EO_CNQ were used). The B2 position on plate 6 resulted in the identification of a BAC which was positive when tested with both primer sets (Figure S10).

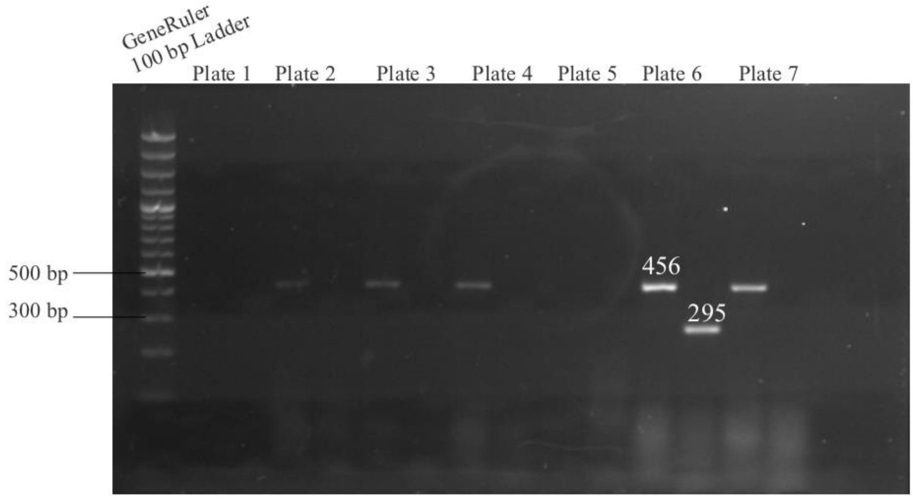

Figure S10. Screening of individual BAC clones from the "*Marinispora*" CNQ-140 BAC library. Whilst many of the clones were positive for the region at the beginning of the gene cluster, only one clone (position B2 on plate number 6) was found to be positive to contain both regions at the beginning and end of the BGC. The 456 bp product was detected in this clone after screening with the EA1 primer set and the 295 bp region was also detected in position B2 on plate six after screening with the EOCNQ primer set.

In order to further confirm that the BAC B2 on plate six was the correct clone, this clone was screened with the PCR primers Clu11, Clu18 and Clu36, and was found to be positive for all three primer sets. As well as testing the BAC with

these primer sets, additional random BACs were also tested as negative controls, to demonstrate that the primers were not randomly annealing to every BAC (Figure S11).

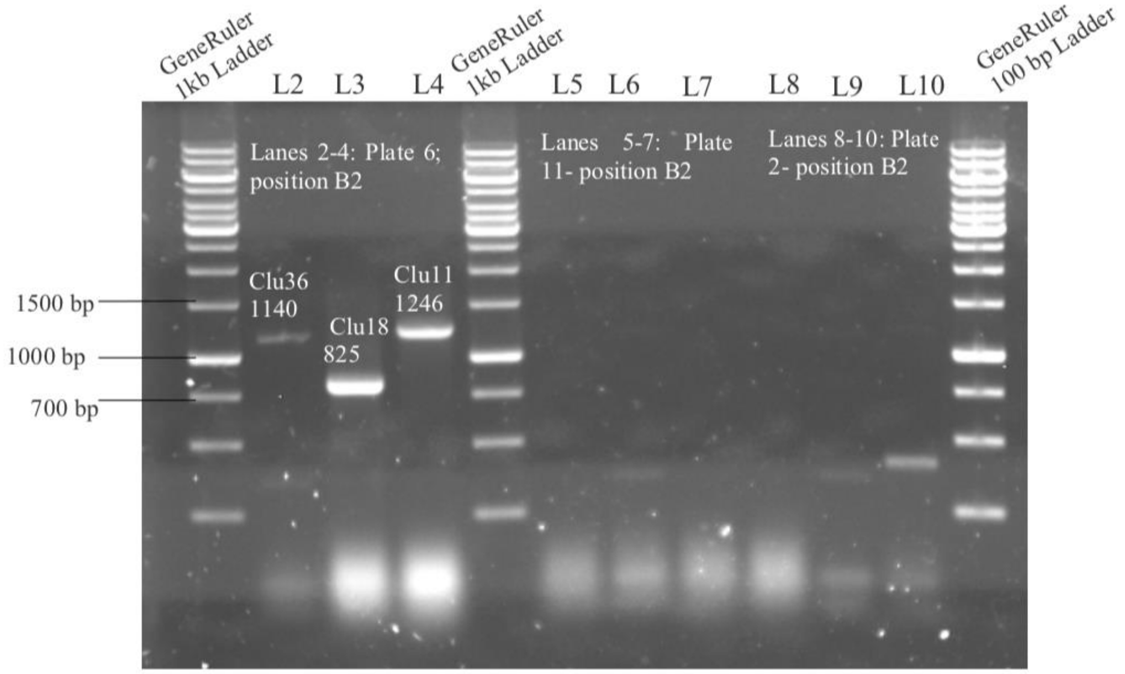

Figure S11. The BAC which was previously found to contain beginning and end regions of the Marinomycin cluster was further screened with three additional primer sets, Clu36, Clu18 and Clu11 (lanes 2-4). The BAC was found to be positive with all three primer sets, giving confidence that this was the correct BAC vector. For negative controls, two additional random BACs were screened with the same primers (lanes 5-10) and were negative, revealing that the primers were not non-specifically annealing to every BAC vector.

After identifying this clone, the BAC library was furthered screened to identify additional BACs which also contained the Marinomycin gene cluster, in order to maximise chances of success. Through further PCR screening as described previously, five additional BAC vectors were found to be positive when screened with primers that amplified regions at the beginning and end of the gene cluster (Figure S12).

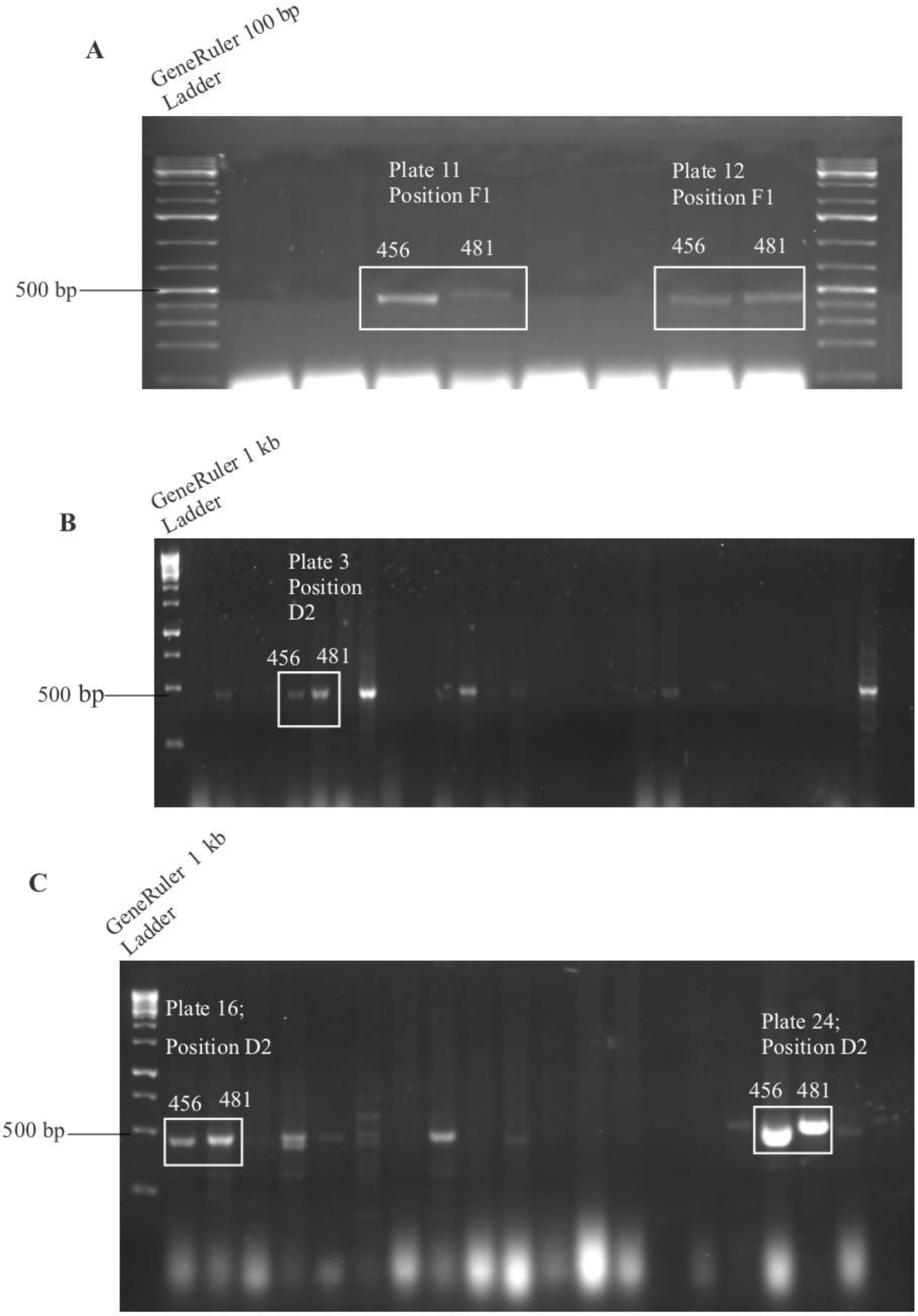

Figure S12. The screening of the "*Marinispora*" CNQ-140 BAC library for additional BACs containing the Marinomycin BGC. The library was screened with primer sets that amplified at the beginning of the BGC (EA1 primer set:456 bp product) and at the end of the cluster (EO_CNQ primer set: 481 bp product). Five additional BAC vectors were found to contain the whole BGC: these are boxed in white. The positive hits were plate 11: position F1, plate 12: position F1, plate 3: position D2, plate 16: position D2 and plate 24: position D2.

4. Testing of Strains for Susceptibility to Marinomycin A

The same overnight starter culture of *S. coelicolor* M1154 was used to inoculate new 2 mL ISP2 cultures within a 24-deep well plate. Purified Marinomycin A was added to the cultures at varying concentrations. No Marinomycin was added to positive control cultures, and negative control cultures contained the same concentrations of rifamycin, as *S. coelicolor* is known to be susceptible to this antibiotic. After twenty-four hours of growth, the OD_{600} values of the cultures were measured (Table S2).

Table S2. OD600 values of *Streptomyces coelicolor* cultures supplemented with antibiotics

Culture Number	Antibiotic added mg/mL	OD_{600}
1	-	2.436
2	-	2.556
3	-	2.439
4	0.05	1.941
5	0.025	2.344
6	0.0175	2.508
7	0.05	0.150
8	0.025	0.152
9	0.0175	0.175

5. Growth and Analysis of Heterologous Host and Marinomycin Production

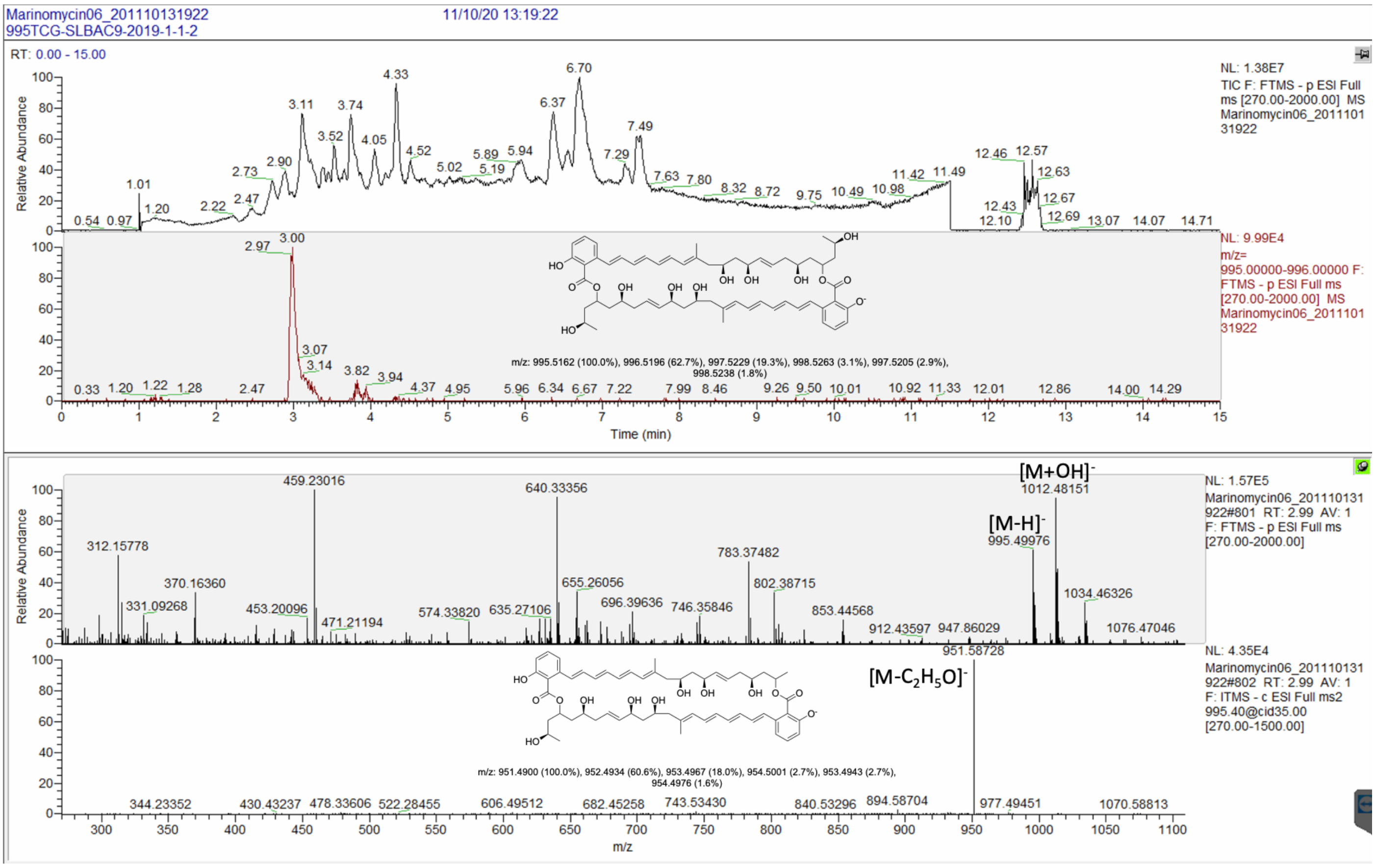

Figure S13. LC-MSMS data for the supernatant of *S. lividans* mutant containing the BGC for marinomycin

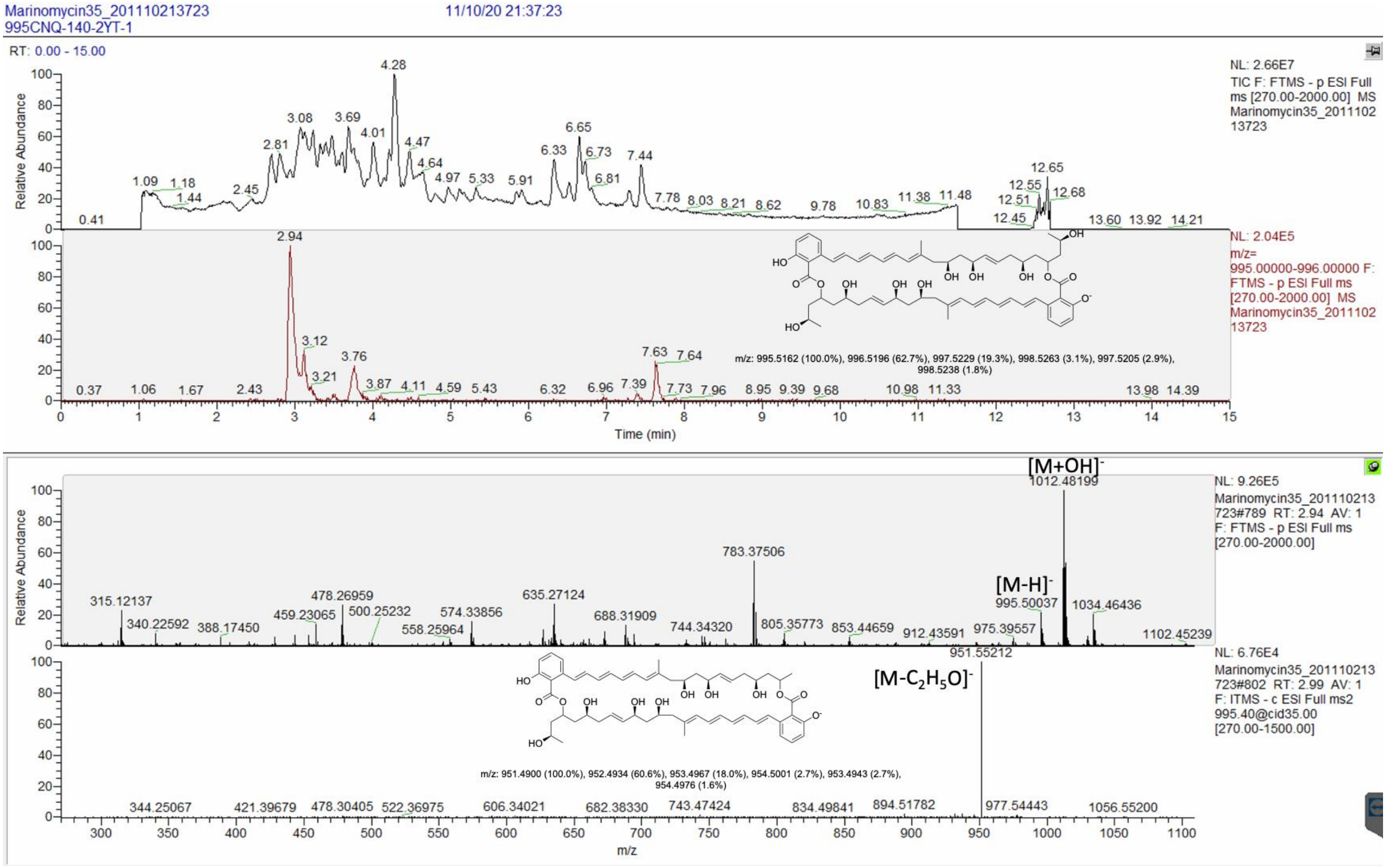

Figure S14. LC-MSMS data for the supernatant of the wild type *Marinospora* CNQ-140

6. Feeding of propionate, 3-fluoropropionate, 4-fluorobutyrate to the WT and heterologous expression strain do not give rise to any observable further products

The natural starter unit for Marinomycin is acetate. Polyketide synthases often exhibit a level of flexibility as to the starter units that they can accept. The sodium salts of propionate, fluoropropionate and 4-fluoropropionate (a highly toxic metabolite, and citric acid cycle inhibitor, was not administered) were administered to 4 day old cultures of both the WT and heterologously expressed strains at a final concentration of 7mM, each in triplicate. Cultures were extracted, as per the standard protocol, and analysed by LC-HRMS. No incorporation whatsoever could be observed, indicating that the Marinomycin loading module is strict in the starter units that it enables to be processed.

7. MinION Sequencing and Analysis

Initial 454 sequence data was obtained in 2012. This was followed, at a later date, by longer read sequence analysis. A further round of sequencing to obtain higher quality sequence data with longer reads was carried out using MinION nanopore. 239 Mb through-put was obtained with an N50 value of 3.9 kb and approx. 40-fold genome coverage. This data verified a GC-content of 74%. The MinION nanopore reads were assembled using Canu and the assembly was then polished using the previously acquired 454-sequencing reads which were aligned onto the Canu assembly using Burrows-Wheeler Aligner providing 8 contigs with a total genome size predicted to be approximately 7.5 Mb. There were no plasmids detected during the genomic analysis.

Gene	Deduced role	ORF size	SIA analogue (Similarity/Identity, %/%)	Protein homolog (Similarity/Identity, %/%)
marA	Beta-ketoacyl-ACP synthase	443	*siaA* (76/68)	*Streptomyces buecherae* WP_187065110.1 (80/72)
marB2	Acyltransferase	322	*siaB2* (75/63)	*Streptomyces buecherae* WP_176160487.1 (77/66)
marB1	ACP S malonyltransferase	774	*siaB1* (82/72)	*Streptomyces buecherae* WP_217201342.1 (81/72)
marC	Enoyl-CoA hydratase	272	*siaC* (84/74)	*Streptomyces buecherae* WP_187060386.1 (86/79)
marD	Polyketide synthase (KS-KR-ACP-KS-DH)	2533	*siaD* (61/51)	*Streptomyces anulatus* WP_228182535.1 (63/52)
marE	Polyketide synthase (KR-ACP-KS-UN-ACP-KS)	2333	*siaE* (70/62)	*Streptomyces buecherae* WP_217201345.1 (74/66)
marF	Polyketide synthase (ACP-KS-DH-KR-ACP-KS-ACP-KS-ACP-KS-ACP-ACP-ACP)	4690	*siaF* (71/62)	*Streptomyces buecherae* WP_217201347.1 (76/68)
marG	Polyketide synthase (KS-DH-KR-ACP-ACP-KS-DH-KR-ACP-KS-DH)	4044	*siaG* (76/68)	*Streptomyces buecherae* WP_176160480.1 (78/71)
marH	Polyketide synthase (KR-ACP-KS-ACP-ACP-KS-ACP-KR)	2775	*siaH* (74/65)	*Streptomyces buecherae* WP_217201351.1 (76/68)
marI	Polyketide synthase (KS-AT-DH-ACP-TE)	1644	*siaI* (81/72)	*Streptomyces buecherae* WP_187064536.1 (85/78)
marJ	3-hydroxy-3-methylglutaryl-ACP synthase	421	*siaJ* (87/75)	*Streptomyces buecherae* WP_176160477.1 (88/78)
marK	Enoyl CoA hydratase	253	*siaK* (82/75)	*Streptomyces buecherae* WP_176165846.1 (89/81)
marL	Acyl carrier protein	81	*siaL* (83/72)	*Streptomyces buecherae* WP_176160476.1 (88/81)
marM	3-ketoacyl-ACP reductase	248	*siaM* (93/84)	*Streptomyces sindenensis* WP_189527439.1 (93/84)
marN	ATP-binding protein	141	NA	*Streptomyces sp.* NBRC 109706 WP_062213218.1 (83/79)
marO	RNA-binding transcriptional accessory protein	828	NA	*Streptomyces triticirhizae* WP_122399972.1 (93/91)
marP	Acyl-ACP desaturase	300	NA	*Streptomyces* WP_139667602.1 (95/90)

marQ	ABC transporter ATP-binding protein	558	*siaO* (47/28)	*Streptomyces sedi* WP_139648298.1 (94/86)
marR	ABC transporter ATP-binding protein/permease	588	*siaT* (47/33)	*Streptomyces triticirhizae* WP_122399519.1(97/94)

Table S3. Analysis of the Marinomycin BGC

Comparison with the Mmarinomycin and SIA7248 clusters indicate that the genes and their organisation are very similar. Modules 3, 4, 6-8, and 10-14 are very highly conserved. The genes with the greatest differences were at the beginning of the biosynthetic pathway, reflecting a difference in the biosynthesis. In the SIA7248 biosynthesis, *sia*D uses a glycerol-derived lactate as the starter unit which differs from Marinomycins biosynthesis which is proposed to use an acetate starter unit.

As with *siaD-siaH* or *marD-marH* (encoding modules 1-13 of the SIA7248 and marinomycin biosynthetic machineries, there are no module embedded AT domains. *siaB1* has been shown to encode a protein which acts as an AT domain as it iteratively loads malonyl-CoA extender units in *trans* onto the ACPs of *siaE-siaH*. A second protein encoded by *sia*B2 is lacking the GHSxG motif required for the acylation of ACPs and is classified as a proof-reading AT. Similarly, *marB2* contains the active-site motif GHSxG and the substrate-binding motif AFHS, specific for malonyl-CoA; while *mar*B1 lacks both motifs. The bioinformatics analysis results suggest that *marB1* and *marB2* share similar roles as *siaB1* and *siaB2* in the biosynthesis of SIA7248, acting as an AT domain and a proof-reading AT, respectively. *siaA*, *siaC*, and *siaJ-L* encoded for an enzyme cassette that installs the β-methyl group at C_{23} in SIA7248. These have 68-75% identity to *marA*, *marC*, and *marJ-L* in the proposed Marinomycin BGC, which is indicated to install the β-methyl group at C_{22} in Marinomycin.

Modules 3, 4, 6, 7, 8, 10-14 of Marinomycin are same as the corresponding modules of SIA7248. Modules 3, 4, 6 and 8 of both Marinomycin and SIA7248 lack embedded ketoreductases. *siaM* has been demonstrated to encode for a β-ketoacyl-ACP reductase which iteratively reduces the β-ketoacyl intermediates tethered to *siaF*. In analogy *marM* (with 84% identity to *siaM*) may also be considered to operate as a *trans*-KR.

The module 9 of Marinomycin contains one additional ACP domain, which also has the signature GxxS motif. Both ACP domains are active and may operate in parallel to increase product turnover.

Comparing to the module 5 in SIA7248, module 5 in Marinomycin BGC contains an active DH domain, generating the C44, C45 double bond, which is the main difference between the two compounds.

The major difference between the SIA7248 and Marinomycin BGCs were found in the first two modules. In the SIA7248 BGC, the first module is a loading module, containing DH-KR-FtbH-ACP. Whilst in the Marinomycin BGC, module 1 contains KS-KR-ACP domains. Phylogenetic analysis revealed that the KS domain in module 1 of the Marinomycin BGC (marD_KS1) could be grouped into a clade with the first KS in the biosynthesis of anthracimycin (Atc), disorazol (Dis) and macrolactin (Mln). These three PKSs lack a loading module, suggesting that the Marinomycin BGC also lacks a loading module as well and module 1 initiates the chain elongation. In this case, to produce Marinomycin similar to SIA7248, using a BGC containing one more extension module than SIA7248 BGC, there must be a redundant module which is not active during the biosynthesis process. Module 2 is highly

possible to be the redundant module, as at least two domains in it, the DH and KR domains, have variations in the active sites, which might inactivate the enzymes.

The MinION 9.4 flow cell was used for microbial genome sequencing and the library was prepared with the 1D rapid ligation kit, as per the manufacturer's instructions. After sequencing the genomes, the data was first analysed using Poretools168. This was first used to convert fast5 files to fasta files, allowing for further processing (poretools fastq test_data/*.fast5). Poretools was then used as a primary analysis tool, for example for determining statistics including the mean read length and maximum length (the command 'stats' generated the read size statistics for a set of fast5 files).

Canu169 was then used for the correction, trimming and assembly of nanopore sequences. This allowed for the generation of contigs from the nanopore data (example command: CANU/canu/*/bin/canu -p CANU_3RW5 -d CANU_3RW5_folder genomeSize=4.0m - nanopore-raw raw.reads.unsorted.3RW5). Aftewards, the Circlator tool170 was used to identify and trim overhangs on chromosomes and plasmids. The Circlator parameters were relaxed to take into account the poorer quality of the nanopore reads, as the default was set to the accuracy expected with PacBio sequencing.

(Example command: circlator all --threads 8 –merge_min_id 83 –merge_breaklen 1000 -- verbose CANU_3RW5_attempt2.contigs.fasta CANU_3RW5_attempt2.correctedReads.fasta.gz circlator_outdir).

This resulted in the finished assembly of genomes using purely MinION nanopore data. However, when Illumina reads were combined to improve the assemblies, the Burrows- Wheeler Aligner (BWA) and Samtools were used together to align and sort Illumina reads to the Canu assembly, to generate a BAM file (First index the files: bwa index contig1.fasta; Second align the files: bwa mem -t 8 contig1.fasta illumina1.fastq.gz | samtools sort > aln.bam).

SPAdes172 was then used to identify any smaller plasmids that may have been missed by the longer nanopore reads. To do this BWA was used to align the Illumina reads to the Canu assembly. Samtools was used to extract unmapped reads, SPAdes was used to assemble unmapped reads. The reads were then trimmed using Samtools.

Next, Pilon174 was used to improve the assembly with the Illumina reads. To do this, alignments of Illumina reads to the Canu assembly were generated, sorted and indexed (BWA and Samtools). Pilon was then run to perform the final assembly (perl pilon –genome genome.fasta –frags aln.bam –output pilon1 –fix all –mindepth 0.5 –changes –verbose –threads 8).

The genome was then submitted to the online RAST248 server for annotation and then to antiSMASH for secondary metabolite BGC detection. This generated 8 contigs shown in Table S4.

Contig	Length (bp)
**1**	4315683
**2**	1878162
**3**	861431
**4**	384583

**5**	26333
**6**	9474
**7**	6830
**8**	2964

Table S4. Contig lengths for the genome sequencing of "*Marinispora*" CNQ-140

8. Alignments and Branching Analysis

Clustal Omega was used to align amino acid sequences and compare the conserved active sites in domains. The iTOL (version 6.0) was used to create the phylogenetic tree of KS domains. All the other KS sequences were selected from the work of Alt and Wilkinson published in 2015.

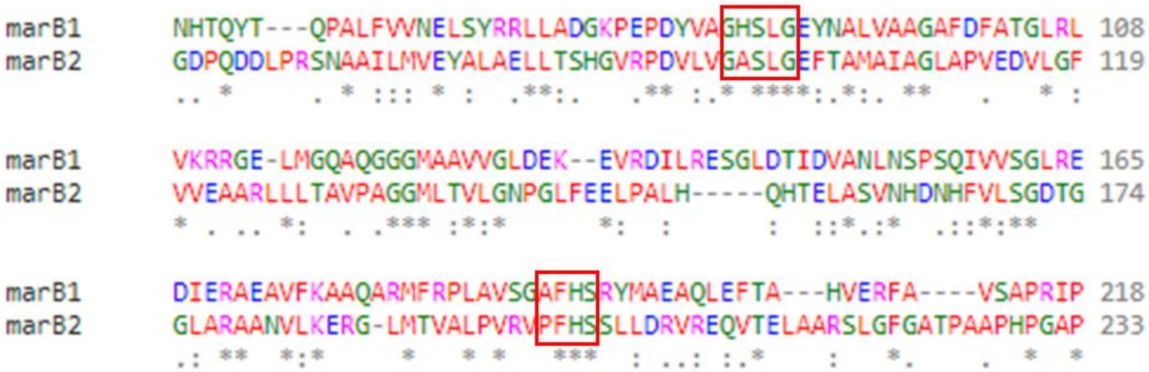

Figure S15. Comparation of active sites in *marB1* and *marB2*.

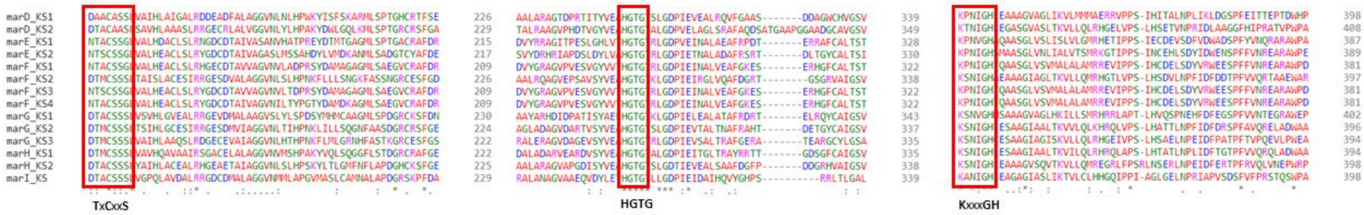

Figure S16. Comparation of KS domains

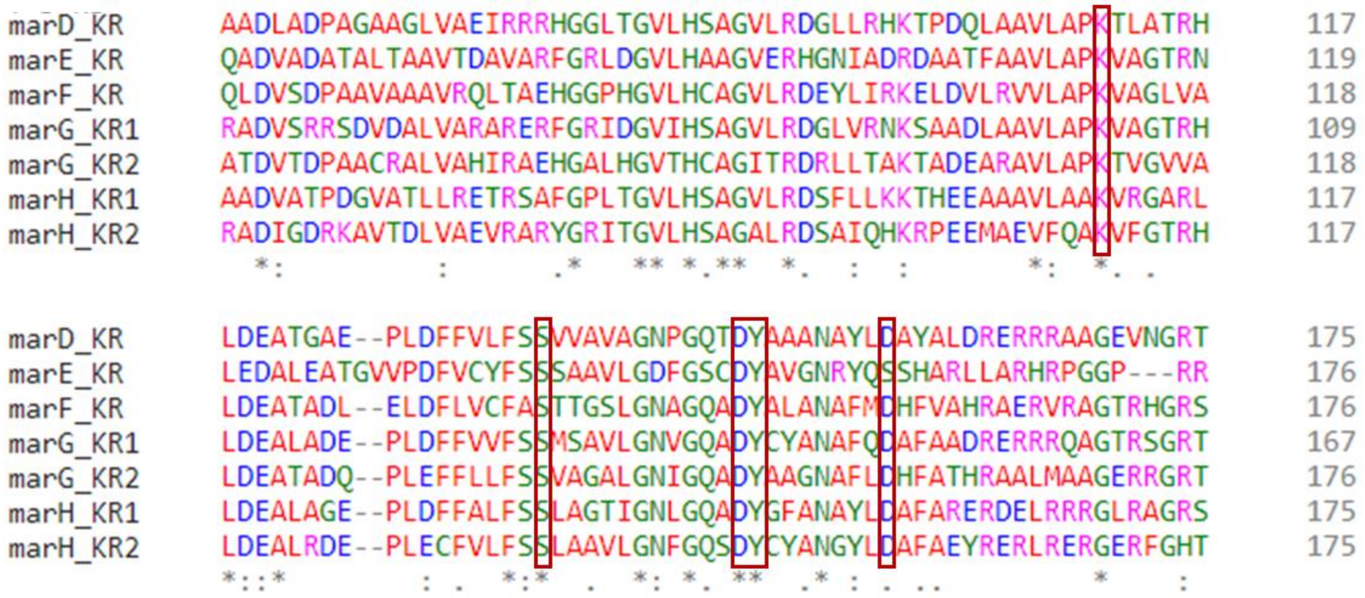

Figure S17. Comparation of KR domains

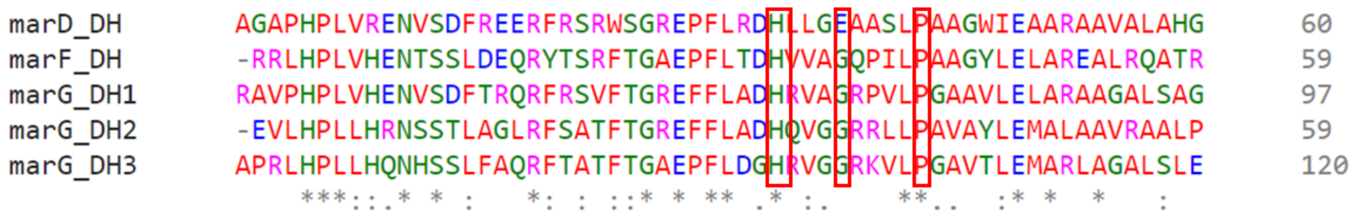

Figure S18. Comparation of DH domains

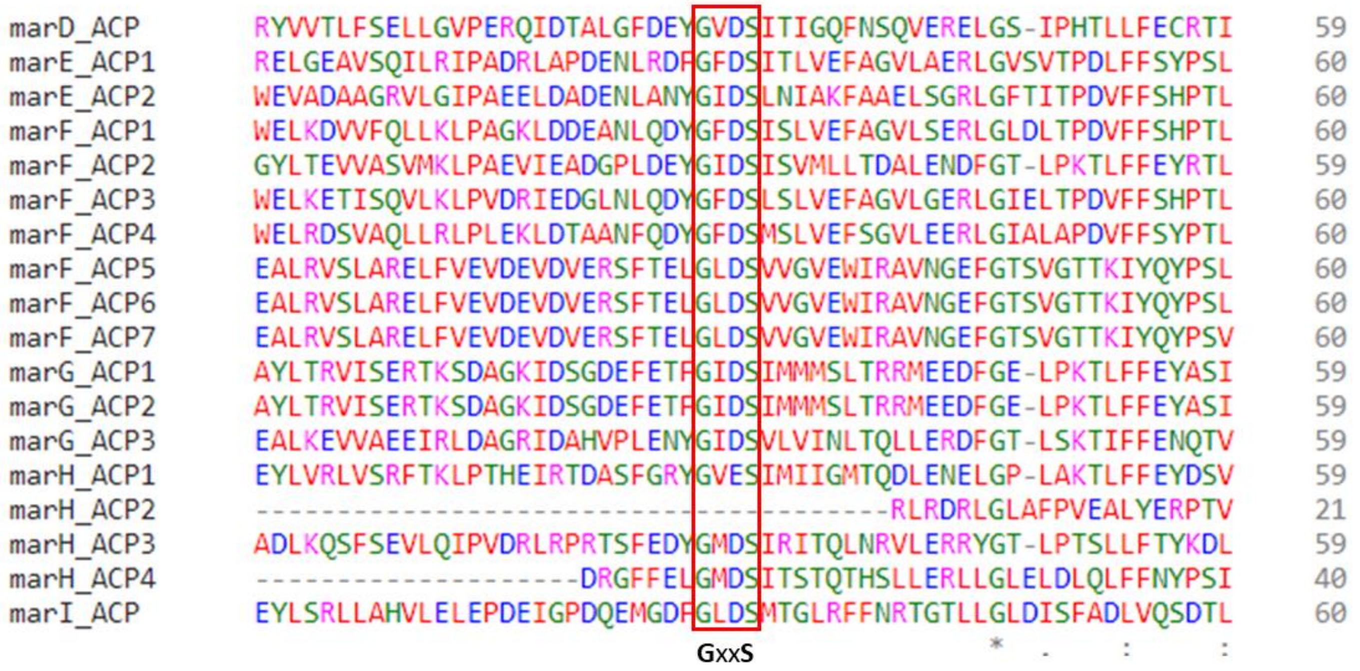

Figure S19. Comparation of ACP domains

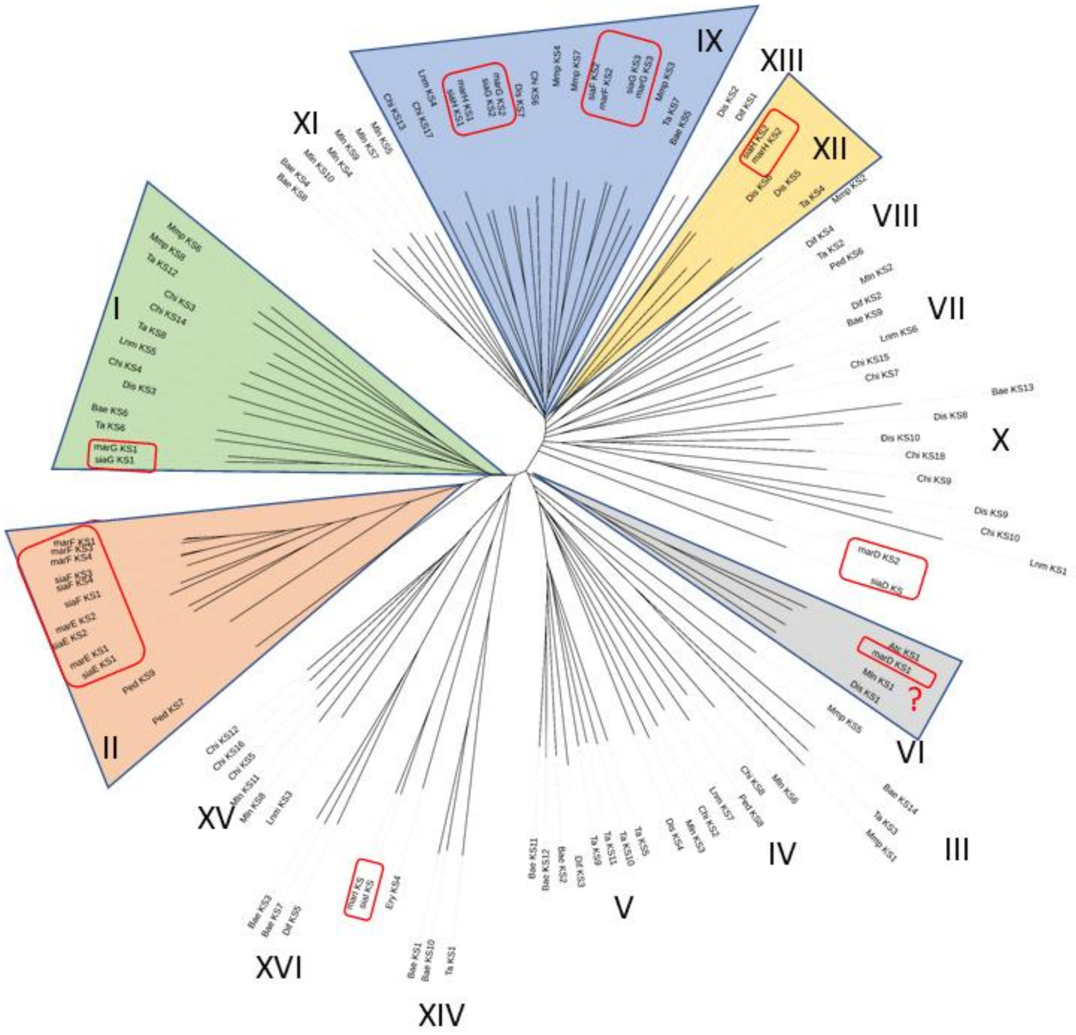

Figure S20. Phylogenetic tree of KS domains. The KS domain in the module 1 was grouped into an unknown clade with Mln_KS1, Dis_KS1 and Act_KS1.

Appendix

1. *Streptomyces lividans*:CNQ marinomycin A deactivation experiment raw data (EIC peak area)

		S. lividans								
		supernatant			lysate			Pellet		
	supernatant	654217	1082194	936857	171469	228807	221937	0	552266	443276
	lysate	0	0	0	0	0	0			
CNQ	pellet	869805	308478	761950						

2. *Streptomyces sp.*:CNQ marinomycin A deactivation experiment raw data (EIC peak area) (L: lysate) (S: supernatant)

Sample ID	Species	Strep:CNQ	Marinomycin A

A1	*S. lividans*	L:s	202485
A2	*S. lividans*	L:s	121768
A3	*S. lividans*	L:s	238804
B1	*S. lividans*	S:S	231991*
B2	*S. lividans*	S:S	32980
B3	*S. lividans*	S:S	23154
C1	*S. coelicolor*	L:s	917312*
C2	*S. coelicolor*	L:s	1434454
C3	*S. coelicolor*	L:s	1554204
D1	*S. coelicolor*	S:S	390190
D2	*S. coelicolor*	S:S	51878
D3	*S. coelicolor*	S:S	0*
E1	FR008	L:s	1918133
E2	FR009	L:s	1446135
E3	FR010	L:s	949939*
F1	FR011	S:S	586440
F2	FR012	S:S	1059121
F3	FR013	S:S	2602307
G1	LQ3	L:s	520948
G2	LQ4	L:s	522458
G3	LQ5	L:s	378183
H1	LQ6	S:S	645801
H2	LQ7	S:S	1383787
H3	LQ8	S:S	328217
liv9-2019S	Het exp.	Supernatant	0
liv9-2019L	Het exp.	Lysate	0
CNQS	CNQ	Supernatant	12705668
CNQL	CNQ	Lysate	6463174
